# Effect of Silane Treatment on Mechanical Properties of Polyurethane/Mesoscopic Fly Ash Composites

**DOI:** 10.3390/polym11040741

**Published:** 2019-04-24

**Authors:** Chuanrui Qin, Wei Lu, Zhenglong He, Guansheng Qi, Jinliang Li, Xiangming Hu

**Affiliations:** 1 State Key Laboratory of Mining Disaster Prevention and Control Co-founded by Shandong Province and Ministry of Science and Technology, Shandong University of Science and Technology, Qingdao 266590, China; qinchuanrui123@163.com (C.Q.); qiguansheng@126.com (G.Q.); lijinliang2008@163.com (J.L.); xiangming0727@163.com (X.H.); 2 College of Mining and Safety Engineering, Shandong University of Science and Technology, Qingdao 266590, China; 3 National Demonstration Center for Experimental Mining Engineering Education, Shandong University of Science and Technology, Qingdao 266590, China

**Keywords:** polyurethane, mesoscopic fly ash, mechanical properties, modification

## Abstract

In view of the accidents such as rock mass breakage, roof fall and coal slide in coal mines, polyurethane/mesoscopic fly ash (PU/MFA) reinforcement materials were produced from polymethylene polyphenylene isocyanate (PAPI), the polyether polyol, flame retardant, and MFA using stannous octanate as a catalyst. 3-Glycidoxypropyltrimethoxysilane (GPTMS) was grafted on MFA surface, aiming to improve the mechanical properties of PU/MFA composites. The analyses of infrared spectroscopy and compression resistance reveal that the GPTMS can be successfully attached to the surface of MFA, and the optimum modification dosage of GPTMS to MFA is 2.5 wt. % (weight percent). On this basis, the effect of GPTMS on the mechanical properties of PU/MFA reinforcement materials during the curing process was systematically investigated through a compression test, a fracture toughness test, a three-point bending test, a bond property test, and a dynamic mechanics analysis. The results show that the compression property, fracture toughness, maximum flexural strength, and bond strength of PU/MFA composites increase by 21.6%, 10.1%, 8.8%, and 19.3%, respectively, compared with the values before the modification. Furthermore, the analyses of scanning electron microscope and dynamic mechanics suggest that the coupling agent GPTMS can successfully improve the mechanical properties of PU/MFA composites because it eliminates the stress concentration and exerts a positive effect on the crosslink density and hardness of PU/MFA composites.

## 1. Introduction

China boasts abundant coal resources, and its annual amount of coal production is increasing year by year. However, due to the complicated geological conditions and harsh working environment in the underground coal mines, and respectively, coal-rock mass tends to become loose during roadway excavation and mining. Loose coal-rock mass can easily cause accidents such as collapse of working face, coal slide of fractured rock mass, gas outburst, and water leakage, seriously affecting the safety of underground workers and the efficiency of coal mining. As a result, researches on grouting reinforcement materials for coal mines have been gradually carried out and have achieved fruitful results after decades of application and development [1,2]. Due to its low viscosity, good fluidity, and controllable curing time, grouting reinforcement materials are generally added to the fractured surrounding rock or loose rock mass. After they become gelled and solidified in a short time, a netted consolidation and a complete force body will be formed from the original fractured and loose rock mass, so that the rock body is capable of bearing surrounding stress effectively [3].

Based on the above grouting reinforcement mechanism, inorganic grouting materials such as water glass, cement, and their mixture have been adopted. While these cheap, non-toxic, flame-inhibitory, and accessible inorganic grouting materials are able to achieve strong strength of the consolidated body, they can only be applied in limited fields because of their unstable curing time, small controllable range and weak toughness [4]. Subsequently, polymers such as acrylamide [5], epoxy resin [6], urea-formaldehyde resin [7], and methyl methacrylate [8] have been used as grouting materials. While these materials solve some problems, which inorganic grouting materials fail to work out to some extent, they can hardly be extensively utilized in fields like coal mines because they are costly and unable to inhibit spontaneous combustion after curing. Given their low viscosity, high activity, controllable curing time, sound toughness, and strength of consolation, polyurethane (PU) reinforcement materials have been widely accepted and used [9]. When the PU slurry is injected into the crevice of the coal-rock mass, the isocyanate group in the slurry can build the chain extension and crosslink reactions with the moisture on the surface of the crevice, forming a gel-like consolation with a certain intensity. In addition, the high-polarity NCO functional group can maintain powerful bond strengths between the fracture surface and the mineral particles of coal-rock mass. Furthermore, in the closed space system, a great internal pressure difference brought about by the CO_2_ released from the reaction can promote diffusion of slurry into deep pores and fractures of the coal seams and enhance the effect of grouting reinforcement [10]. Unfortunately, PU reinforcement materials also face the problems of high cost and combustibility. In hope of solving these problems, the researchers added inorganic fillers such as cement [11], water glass [12], and MFA [13] into the organic matrix to form organic-inorganic composites. Compared with the pure organic materials, these composites succeed in overcoming the abovementioned problems. Among them, mesoscopic fly ash (MFA), the waste material left by industrial coal combustion, boasts stable chemical properties and cheap and accessible raw materials. When it is filled into the organic matrix as the filler, it will not only act as the dispersed phase to enhance the pressure bearing capacity of the material and effectively reduce the cost, but also eliminate the solid waste and achieve reutilization so as to avoid environmental pollution [14]. Liu et al. [15] took MFA and PU as basic materials to prepare a new type of polymer concrete reinforcement material, which was significantly better in terms of its strength and curing time compared with ordinary concrete materials. Rahman et al. [16] prepared a type of PU/OFA composite coating material by the method of in-situ polymerization. They found that the mechanical strength, bond strength and moisture resistance of the composite coatings could be effectively improved when the content of OFA was greater than 1 wt %. It can be known that for mesoscale inorganic particles, the properties of the material may change significantly and differ completely from their bulk counterparts. As the size of the material decreases, both the proportion of surface atoms and the reactivity of the material increase. As a result, the material becomes a highly reactive catalyst in which the surface atoms serve as active centers for elementary catalytic processes [17]. Therefore, thanks to its unique properties, the inorganic MFA is able to disperse in PU matrix to form the composites, which further improves the physical and chemical properties of organic matrix. However, the dimensional particles tend to undergo agglomeration followed by insufficient dispersal in the polymer matrix. Meanwhile, since the surface properties of inorganic MFA particles differ from those of organic matrix, to simply mix MFA with organic matrix tends to result in poor affinity between them, thus affecting the dispersion of MFA particles in the organic matrix. As a result, stress may concentrate to a certain extent, and mechanical properties of the composites may decrease [18,19,20]. Hence, researchers have proposed specific methods of promoting the dispersion and crosslink in the organic matrix by pretreating the inorganic MFA particles [21,22].

As a compound with amphiphilic functional groups, silane coupling agent can react with both inorganic and organic substances and form “chemical bridges” to alter the interfacial adhesion between them. Besides, it can also improve the compatibility between the inorganic mesoscale particles and polymer matrix and thus greatly enhance the distribution of inorganic mesoscale particles in the polymer matrix [23].

Hence, GPTMS was grafted on MFA surface by the surface modified technique. The modified MFA was then mixed with PU matrix to obtain PU/MFA composites. The optimal modification dosage of GPTMS was investigated, based on which the mechanical properties (compression, fracture, bending, bond and dynamic mechanics) of the modified PU/MFA composites during the curing process were studied. Moreover, the mechanism for the coupling agent GPTMS to effectively improve the internal crosslink density and hardness of the composites without causing stress concentration was proposed.

## 2. Experiments

### 2.1. Components of Experimental Materials

In this study, the PU/MFA composites prepared are dual-component organic/inorganic mixtures whose components are listed in Table 1. (All PU/MFA specimens were measured by dibutylamine method, *R* values range from 1.0 to 1.05)

### 2.2. Optimization of GPTMS Dosage

In order to obtain the optimum amount of the coupling agent GPTMS, the compressive strength of PU/MFA composites modified by different contents of GPTMS (0.5, 1.5, 2.5, and 3.5 wt. %) was investigated through a series of optimization experiments. The MFA was modified with different dosages of GPTMS in accordance with the following steps: First, 500 g of MFA was mixed with 200 mL of ethanol aqueous solution at a volume ratio of 9:1. Next, different dosages of GPTMS were added in order and then stirred at the speed of 600 rpm for 2 h. Finally, the MFA was dried under the vacuum environment of 50 °C for 24 h, after which it was taken out and sealed for storage.

### 2.3. Preparation for Specimens

**Figure 1:**
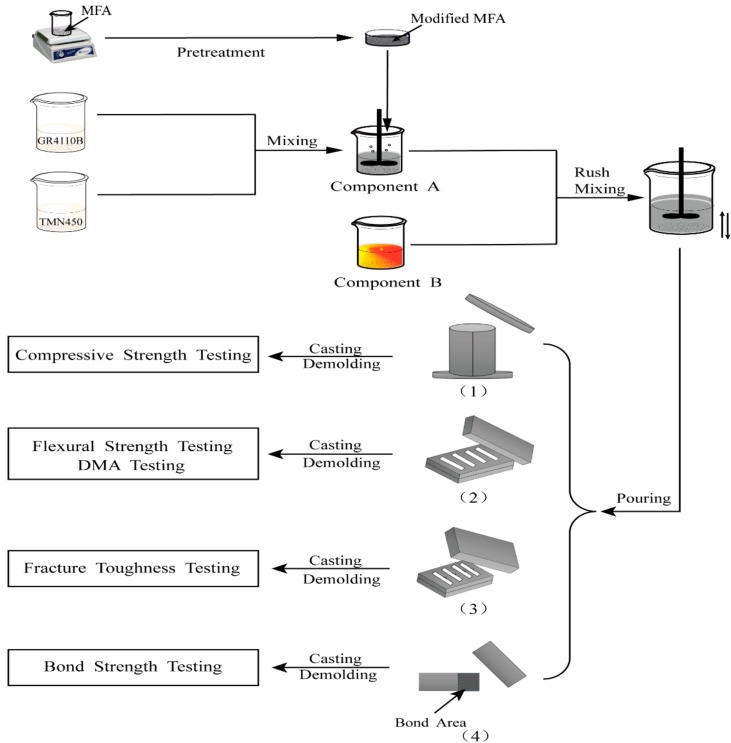
Schematic diagram of PU/MFA composites preparation.

**Figure 2:**
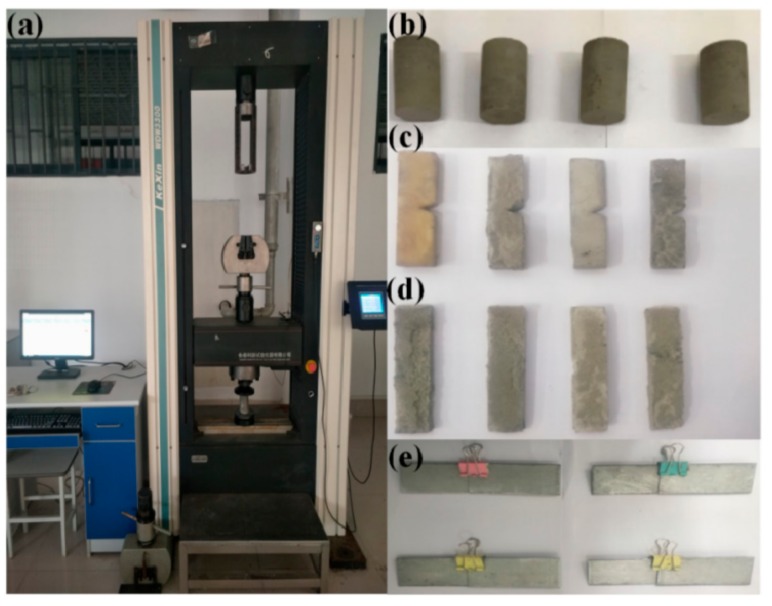
Electronic Universal Testing Machine (**a**) and Measuring Specimens: (**b**) Cylindrical Specimens, (**c**) Three-point Bending/DMA Specimens, (**d**) Rectangular Specimens, and (**e**) Bond Specimens of Iron Batten.

**Figure 3:**
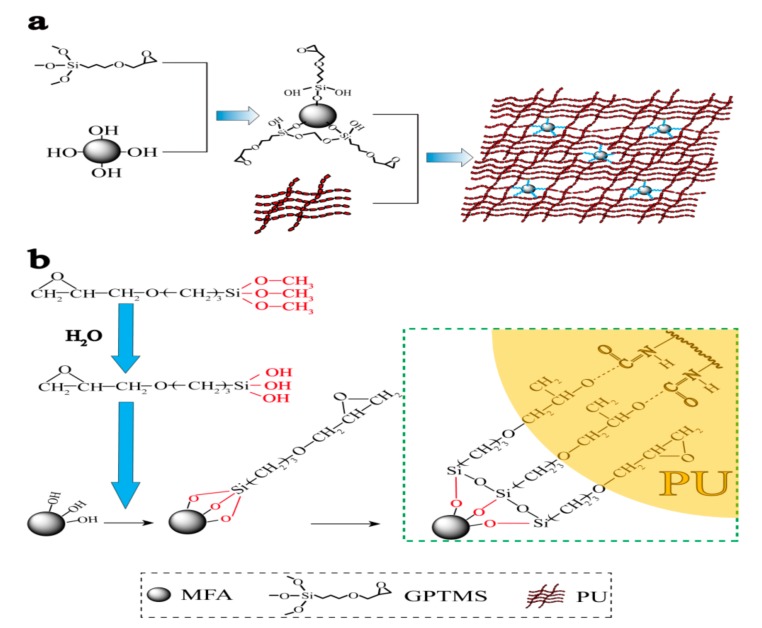
Schematic illustration of the synthesis of PU/MFA composites (**a**) and the crosslink mechanism of modified MFA by 3-Glycidoxypropyltrimethoxysilane (GPTMS) and PU matrix (**b**).

**Figure 4:**
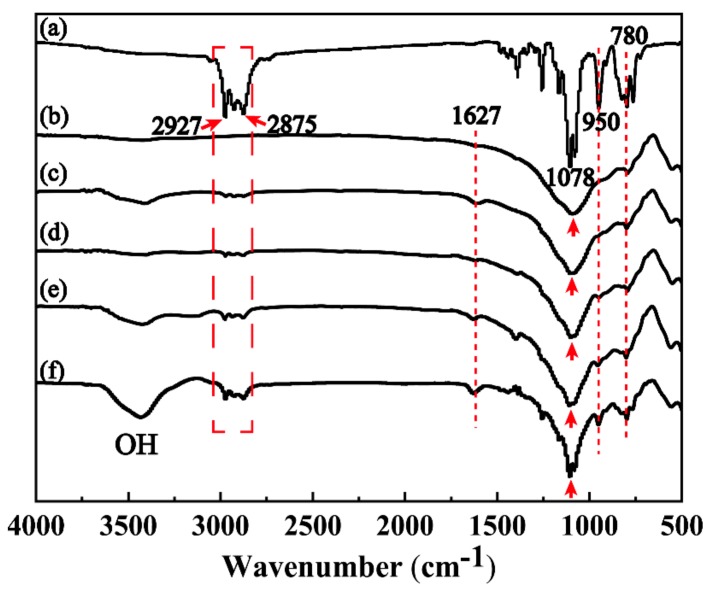
Infrared spectra of coupling agents of GPTMS, unmodified MFA and MFA modified by different contents of GPTMS: (**a**) GPTMS; (**b**) unmodified MFA; (**c**) MFA modified by 0.5 wt. % GPTMS; (**d**) MFA modified by 1.5 wt. % GPTMS; and (**e**) MFA modified by 2.5 wt. % GPTMS; (**f**) MFA modified by 3.5 wt. % GPTMS.

The schematic diagram of PU/MFA composites preparation is shown in Figure 1. First, abovementioned modified MFA was weighed and evenly mixed with polyether polyol GR4110B and TMN450. Then, proper amounts of the flame retardant TCPP and the catalyst stannous octoate were added in turn. After being fully stirring at the speed of 600 rpm, it was recorded as Component A. The PAPI, recorded as Component B, was mixed with Component A and then stirred evenly at the speed of 600 rpm for 30 s. Next, they were, respectively, poured into molds that had been evenly coated with silicone oil for 1 h (as displayed in Figure 1, (1)–(4)). After full consolation, the cylindrical specimens with a diameter of 50 mm and a height of 100 mm were taken from the mold and solidified for different times (1, 3, 7, 14 and 28 days) under a dry and closed environment of 23 ± 1 °C. Subsequently, compressive strengths of the specimens were tested. Meanwhile, the fully poured rectangular specimen (60 × 10 × 3 mm^3^, length × width × height), the three-point bending specimen (53 × 12 × 6 mm^3^, length × width × height) and the bond area (12.5 ± 0.25 mm) of iron batten (100 × 25 × 1.5 mm^3^, length × width × height) were also solidified for different times (1, 3, 7, 14 and 28 d) under a dry and closed environment of 23 ± 1 °C. The specimens were prepared for a fracture toughness test, a three-point bending test, a dynamic mechanics analysis, and a bond strength test, respectively.

### 2.4. Analyses and Characterization Techniques

Infrared spectroscopy analysis: Characteristic functional groups of the specimens were detected by a Fourier Infrared Spectrometer (Nicolet-8700, Thermo Nicolet Co., Ltd., Federal Way, WA, USA). Infrared spectrum testing was conducted on the MFA modified by different contents of GPTMS (0.5, 1.5, 2.5 and 3.5 wt. %) and the unmodified MFA. The powdered specimens and dry potassium bromide (KBr) powder were mixed at a ratio of 1:150 and compressed into tablets. Changes of peaks in infrared spectrum were observed through scanning, and meanwhile the organic functional groups were analyzed. In the test, the measurement range was set to the wavelength of 500–4000 cm^−1^.

Scanning electron microscope (SEM) analysis: Morphologies of the specimens were observed by a high-magnification SEM (JSM-7800F, Electronic Jeol Co., Ltd., Tokyo, Japan). Under the acceleration voltage of 25 kV, 2.5–5 mm cubes were taken from the treated standard specimens and dried to constant weights. The fracture surfaces of the block specimens were coated with gold in a SBC-12 ion sputtering apparatus.

Dynamic mechanics analysis (DMA): The dynamic mechanical properties of PU/MFA specimens were studied by using a dynamic mechanical analyzer (DMA Q800 of TA instruments, New Castle, DE, USA). The DMA measurements, which adopted rectangular specimens (6 × 10 × 3 mm^3^) and three-point bending configurations (span: 50 mm), were carried out using air as the medium. The DMA spectra, which could reflect changes in the storage modulus *E* and the mechanical loss factor tan δ under the action of temperature (*T_g_*), were measured in the temperature range of 30–180 °C with a heating rate of 5 °C/min at a test frequency of 1 Hz.

Uniaxial compressive strength test (Figure 2): The uniaxial compressive strength was tested by a micro-control electronic universal testing machine (WDW3300, Kexin Laboratory Instruments Co., Ltd., Changchun, China). The PU/MFA reinforcement materials were observed in accordance with the GB/T1041-2008 standard method for the uniaxial compressive strength testing for at room temperature. In the test, the speed of testing machine was 10 mm/min.

Fracture toughness test: The fracture toughness was also tested by the micro-control electronic universal testing machine. The PU/MFA reinforcement materials were observed in accordance with the ISO-13586-1 standard method for the fracture toughness testing at room temperature. In the test, the specimens were loaded at three points along with a 50 mm measuring span, and the speed of the testing machine was 1 mm/min.

Three-point bending test: The three-point bending test was performed by the micro-control electronic universal testing machine. The PU/MFA reinforcement materials were observed in accordance with the EN63 standard test method for the flexural strength testing at room temperature. In the test, the specimens were loaded at three points along with a 50 mm measuring span, and the speed of testing machine was 1 mm/min.

Bond strength test: The bond strength was tested by the micro-control electronic universal testing machine. The PU/MFA reinforcement materials were observed in accordance with the GB/T7124-2008 standard test method for the bond strength testing at room temperature. In the test, the test force changed at the rate of 8.5 MPa/min.

Each of reported values of the physical and mechanical parameters represents the average value of at least five specimens.

## 3. Results and Discussion

### 3.1. Mechanism Analysis

Figure 3 presents the crosslink mechanism of modified MFA by the coupling agent GPTMS and the PU matrix. The modification of MFA particles by GPTMS organizes the surface of MFA particles and enables MFA to uniformly disperse in the PU and densely connect with the PU matrix. In this way, PU/MFA materials with excellent mechanical properties are prepared, as shown in Figure 3a. The surface of granular MFA, which can easily unite water molecules in the air to carry hydroxyl group, shows strong hydrophilic and oleophobic properties. Meanwhile, it is incompatible with the organic matrix, resulting in defects in the material structure. Through the modification treatment by the silane-coupling agent, the surface of MFA changes from hydrophilicity to lipophilicity, which increases the compatibility between the organic phase and inorganic phase and promotes the mechanical properties of PU/MFA composites. As presented in Figure 3b, the basic mechanism can be explained as follows. For one thing, the alkoxy group in the molecule is hydrolyzed to form silanol when the surface of MFA is modified by GPTMS. Meanwhile, the silanol molecules associate with each other to form a reticular membrane covering the surfaces of mesoscale particles. Then, the silanol molecules are dehydrated and condensed with the hydroxyl group on the MFA surface. Thereby, the chemical bond can be formed by crosslink. Accordingly, the surface of MFA exhibits a higher degree of organization and changes from hydrophilicity to lipophilicity. For another thing, the epoxy functional group at the end of GPTMS reacts with the organic matrix through the ring-opening reaction, which promotes the increase of crosslink density of the organic phase. Therefore, GPTMS influences the mechanical properties between MFA and PU matrix significantly.

### 3.2. Modification and Optimization of MFA

The infrared spectra of GPTMS, unmodified MFA and MFA modified by different contents of GPTMS (0.0, 0.5, 1.5, 2.5 and 3.5 wt. %) are presented in Figure 4. It can be observed from Figure 4 that the asymmetric vibration peak of –CH_2_ in the chain segment of glycidyl ether of GPTMS molecule appears in the range of 3000–2800 cm^−1^; the vibration absorption peak of Si−O–Si appears at 1078 cm^−1^; and the absorption peak of C–O–C appears at 950 cm^−1^ (Figure 4a). For MFA, the vibration absorption peak of Si–O–Si appears in the range of 1200–1000 cm^−1^ (Figure 4b). Moreover, the vibration absorption peak of Si–O appears at 780 cm^−1^ in the spectrum, indicating that SiO_2_ is the essential component of MFA [24,25]. Figure 4c–f demonstrate that, as the concentration of MPTMS rises, the vibration absorption peaks of –CH_2,_ Si–O–Si and Si–O are gradually strengthen in the range of 3000–2800 cm^−1^, at 1078 cm^−1^, and at 780 cm^−1^, respectively. Furthermore, the absorption peaks of C=O and C–O–C climb at 627 cm^−1^ and at 950 cm^−1^ synchronously. This is mainly because while GPTMS is modifying MFA, the epoxy group undergoes a ring-opening reaction at the end of GPTMS molecular chain [26,27]. Besides, a comparison of Figure 4b–f suggests that vibration absorption peaks of modified MFA are sharper than those of unmodified MFA. This is probably because with the addition of GPTMS, the two vibration absorption peaks in the molecular chains of MFA and GPTMS superpose in the modification process. Therefore, it can be concluded through the infrared spectroscopy analysis that the surface of MFA particles is gradually organized and well cross-linked with GPTMS molecules. In addition, as the concentration of GPTMS increases, the content of organic functional groups in MFA grows significantly.

### 3.3. Compressive Strength of PU/MFA Composites

**Figure 5:**
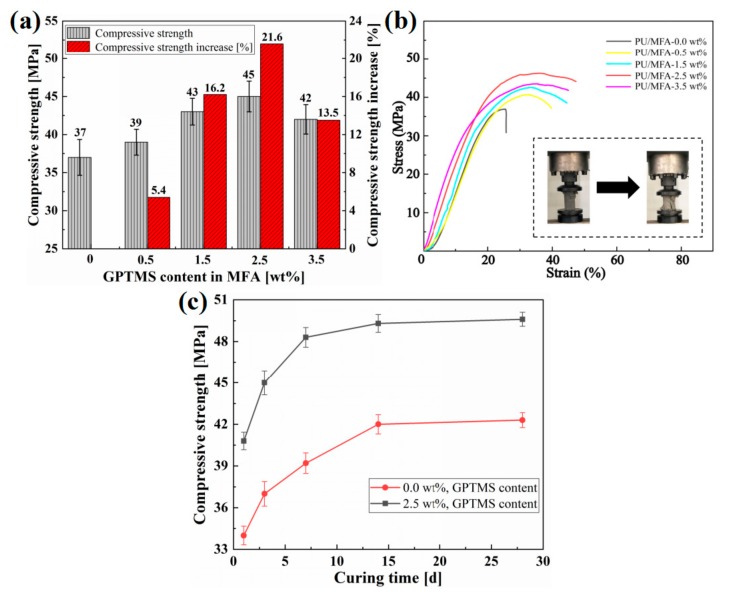
(**a**) Compressive strength of specimens under different GPTMS content in MFA; (**b**) stress-strain curves of PU/MFA composites with different GPTMS contents; and (**c**) compressive strength of -0.0 wt. % and -2.5 wt. % PU/MFA specimens at different curing times.

Compressive strength mainly reflects the mechanical strength of coal-rock mass reinforcement material. Therefore, the study on pressure-bearing capacity of the composites plays a decisive role in enhancing its mechanical support [28]. First, as the GPTMS concentration increases from 0 to 3.5 wt. %, the compressive strength of the PU/MFA composites increases gradually and then decreases, as shown in Figure 5a. When the GPTMS concentration is 2.5 wt. %, the compressive strength of PU/MFA specimen reaches a peak of 45 MPa, 21.6% higher than the value before the modification. Meanwhile, the stress-strain curves of PU/MFA composites with different GPTMS contents during the compression are exhibited in Figure 5b. It can be explained that when GPTMS molecule touches the surface of MFA, an end of the molecule forms a chemical bond with the hydroxyl group on the surface of MFA through chemical reactions such as autolysis, dehydration, and condensation, while the other end binds firmly with PU matrix. As a result, a solid interface layer is greatly enhanced between the two phases. MFA no longer simply disperses in the internal organic PU. Instead, it is tightly cross-linked between the two phases. The property of inorganic filler is fully exerted to promote the pressure-bearing capacity of PU/MFA specimens [29,30]. However, when the GPTMS content increases from 2.5 to 3.5 wt. %, the compressive strengths of the specimens decrease because of excessive GPTMS. This is caused by two reasons. First, the excessive GPTMS results in the formation of silanol molecules on the surface of MFA through constant hydrolysis and condensation between the molecules, and these silanol molecules reduce the crosslink density between PU matrix and GPTMS molecules to some extent. Second, excessive GPTMS weakens the interface bond by forming a thick silane layer prone to adhesive damage in the interface between the two phases. In addition, since the excessive GPTMS molecule is prone to self-polymerization, it also reduces the reactivity between modified MFA and organic PU matrix [31]. Thus, the two effects both have negative impacts on the compressive strength of PU/MFA materials. Consequently, when 3.5 wt. % GPTMS is added, the compressive strength of the PU/MFA composites decrease.

Figure 5c shows the compressive strengths of PU/MFA-0.0 wt. % and PU/MFA-2.5 wt. % specimens at different curing times (1, 3, 7, 14 and 28 days). It can be observed from Figure 5c that their compressive strengths increase rapidly at the first three phases and then increase slowly to stable values as the curing time goes by. The rapid rise of compressive strength may be due to the increase of crosslink density of organic PU matrix. Besides, as the curing time passes, the crosslink densities of the two specimens increase further, and their internal bonding becomes closer, finally achieving stable values upon completion of the curing. Compared with the PU/MFA-0.0 wt. % specimen, the PU/MFA-2.5 wt. % has much higher compressive strength at the same moment. Therefore, the application of GPTMS can improve the strength of PU/MFA in the early stage, as it achieves a uniform distribution and an accelerated curing process by increasing the superficial area and response point of MFA in PU matrix. In the meantime, it also exerts a positive effect on the strength of PU/MFA composites in the later stage [32].

### 3.4. Fracture Toughness and Flexural Strength

**Figure 6:**
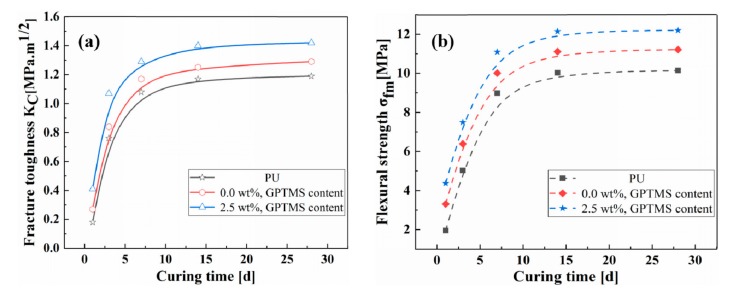
The fracture toughness *K*_c_ (**a**) and the maximum flexural strengths σ_fm_ (**b**) of PU, PU/MFA-0.0 wt. %, and PU/MFA-2.5 wt. % specimens at different curing times.

Figure 6 shows the fracture toughness and flexural strengths of PU, PU/MFA-0.0 wt. %, and PU/MFA-2.5 wt. % specimens at different curing times. Firstly, it can be intuitively seen from Figure 6 that the fracture toughness and flexural strengths of the three specimens are improved rapidly in the first 7 days, due to the increase of crosslink density mentioned above. When the curing time reaches 28 days, the performances of the three specimens tend to stabilize, indicating that they have been completely cured. Compared with pure PU, the flexural strengths of PU/MFA specimens are obviously improved after MFA is mixed with GPTMS, as displayed in Figure 6a. It can be found through further comparison that compared with pure PU, Kc, values of the PU/MFA-0.0 wt. % specimen and the PU/MFA-2.5 wt. % specimen increase by 8.4% and 18.5%, respectively. This indicates that the mix of MFA promotes the fracture toughness of PU/MFA-0.0 wt. % composites. On this basis, the modification of MFA by GPTMS further enhances the fracture toughness of PU/MFA-2.5 wt. % composites. Usually, when the composites are damaged (fractured) by the impact load, the toughness mainly depends on the amount of impact energy absorbed by the material and the resistance to crack growth [33]. Pure PU primarily undergoes brittle fracture. Its fracture surface is generally smooth and the material under the section is not plastically deformed, which requires limited damage energy. On the one hand, after MFA is mixed with the PU matrix as the second phase ductile particles, the crack tip generated by the destruction of the particles and polymer matrix tend to form a plastic deformation zone, causing the crack tip to shield and form a ductile crack bridge from the ductile particle. When compressive stresses are generated on both sides, the particles and the polymer matrix together prevent the outer crack from expanding further, so that the toughness is enhanced [34,35]. On the other hand, it is observed that cracks, which are not flat with cavities, are generated in the polymer matrix and on the fracture surface. This is probably because cracks deflect to a certain extent. They bypass particles and expand in interfaces of particles and PU. While the crack expansion path is lengthened, the resistance, the energy consumption and the toughness get strengthened. In addition, the cavities whose sizes are much larger than those of MFA particles on the fracture surface also raise the toughness of PU/MFA. The mechanism of cavity toughening can be explained as follows. When the material is impacted, the strength of MFA becomes lower than that of PU and is tightly combined under the action of GPTMS. Subjected to the external force, the two specimens release the triaxial tension formed by the contraction resistance of the V-shaped slit, so that the material around the fracture suffers from large-scale plastic deformation [36]. Therefore, the addition of GPTMS greatly improves the fracture toughness of PU/MFA materials.

Figure 6b displays the maximum flexural strengths of pure PU, PU/MFA-0.0 wt. % and PU/MFA-2.5 wt. % specimens at different curing times. The maximum flexural strength of PU/MFA-0.0 wt. % composites on the 28th d increase by 10.6%, compared with that of pure PU. The maximum flexural strength of PU/MFA-2.5 wt. % is 12.2 MPa, which is 8.8% higher than that of PU/MFA-0.0 wt % composites. There are two main reasons for the increase. One is that the filler MFA bonds closely with organic PU matrix. The other is GPTMS organizes the surface of the inorganic MFA filler and uniformly disperses inside the organic phase to avoid the concentration of stress.

### 3.5. Bond Property Analysis

**Figure 7:**
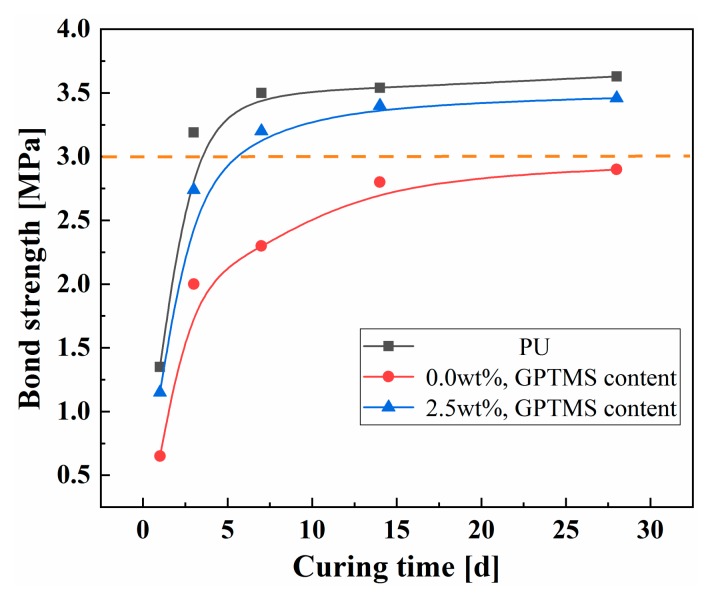
Bond strengths of PU, PU/MFA-0.0 wt. %, and PU/MFA-2.5 wt. % specimens at different curing times.

Figure 7 presents the bond strengths of pure PU, PU/MFA-0.0 wt. %, and PU/MFA-2.5 wt. % specimens at different curing times. It can be seen from Figure 7 that the bond strengths of the three specimens first grow rapidly and then tend to stabilize as the curing time increases. The rapid increases of the bond strengths in the first 7 days are also caused by the rapid crosslink of bonded materials. Besides, unlike the compression, fracture and bending properties, the bond strength of pure PU reaches its greatest value 3.63 MPa on the 28th d. On the contrary, after the proper addition of MFA, that of PU/MFA-0.0 wt. % composites reaches the lowest value 2.9 MPa on the 28th d. The main reason is that after being cured, pure PU is regarded as a kind of thermoset hard material. It is mainly a process of gradual gelation of liquid reactant. At the initial stage of the reaction, PU is formed into a thermoplastic and soluble polymer with the highest bond property. As the reaction proceeds, the molding process ends when the molecular chain between the crosslink points reaches the corresponding length. At this moment, the surface bond strength is almost reduced to zero and mainly depends on the hard segment of pure PU molecular chain [37]. As MFA is incorporated into the interior of the organic PU, its inorganic mesoscale particles act as the hard segment of the organic PU chain to some extent, increasing the Young’s modulus. However, since the interface between the bonding surfaces differs from the organic matrix surface in terms of properties and it is unable to crosslink the surface of MFA, the quantization conditions for forming a chemical bond can hardly be satisfied, so that the growth process of the bond strength stays in the unformed stage of pure PU. Therefore, when the organic PU is filled with inorganic rigid MFA particles, the composites cannot fully bond themselves and the MFA cannot provide partial strength, which reduces the bond properties of the composites to some extent. When the MFA is modified by 2.5 wt. % GPTMS, the bond strength of the PU/MFA-2.5 wt. % specimen increases by 19.3% to 3.46 MPa on the 28th d. This is because when MFA is modified by GPTMS with epoxy group, the end of the molecular chain rapidly reacts with -OH on the surface of MFA to form hydrogen bonds. Under certain conditions, condensation, dehydration and solidification will occur and further form the covalent bond. As the modified MFA filled into the PU matrix, the epoxy group at the other end of the GPTMS is opened and simultaneously connected to the organic PU, thereby promoting the formation of a good interfacial bonding between the MFA-GPTMS-PU and these two interface properties. As a result, materials with large differences are firmly combined [38]. Compared with unmodified PU/MFA composites, the one modified by GPTMS boasts better bonding performance.

### 3.6. Fracture Surface Morphology of PU/MFA Materials

**Figure 8:**
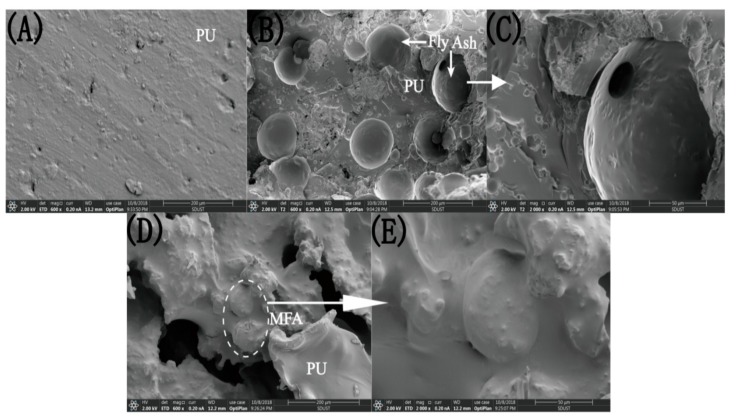
The SEM micrographs of fracture surface of (**A**) PU, (**B**) and (**C**) PU/MFA-0.0 wt. %, (**D**) and (**E**) PU/MFA-2.5 wt. % specimens after fracture toughness test.

Figure 8 shows the SEM micrographs on fracture surfaces of PU, PU/MFA-0.0 wt. % and PU/MFA-2.5 wt. % specimens. The fracture surface of pure PU is smooth and flat, and its structure is uniform without any cells. The fracture belongs to brittle fracture, as illustrated in Figure 8B. After inorganic MFA particles are mixed into the organic PU, they quickly combine with each other to form an organic/inorganic hybrid system. As can be seen from Figure 8B, the MFA particles uniformly distribute in the organic matrix. Its surface is relatively smooth, and the fracture surface of the material is relatively rough. Further, from the partial amplification in Figure 8C, a void can be found at the interface between the PU matrix and the MFA particle, indicating that no chemical crosslink is formed between the MFA and the PU matrix. When the material is subjected to an external force, the stress cannot be effectively transmitted from the PU matrix to the rigid MFA particles, resulting in stress concentration at the interface of the partial phase and a decline in the overall strength of the material. The cross-sectional morphology of the PU/MFA materials modified by 2.5 wt. % GPTMS is shown in Figure 8D. On the one hand, it can be found that the uniformly dispersed surface of MFA is fully coated by organic PU matrix at this time. As can be found from the further enlarged graph in Figure 8E, the interface between the two phases is closely bonded. The addition of GPTMS successfully removes the gap existing at the interface of the two phases and eliminates the discontinuity at the boundary. Accordingly, the surface of MFA is optimized and organized, and the stress concentration is reduced [39]. On the other hand, cracks and a cavity are observed in Figure 8D. When GPTMS is successfully connected to MFA and organic PU, the composites are broken by external force, causing the crack to be deflected. This increases the resistance of crack propagation to some degree. In addition, the cavity region formed when the MFA particles peel off from the organic PU will improve the material toughness. Therefore, both MFA particles and PU may be conducive to improving material toughness. GPTMS, which acts as a crosslink agent, promotes the formation of ductile deformation and toughness-improving zone in a wide range [40].

### 3.7. Dynamic Mechanical Analysis

**Figure 9:**
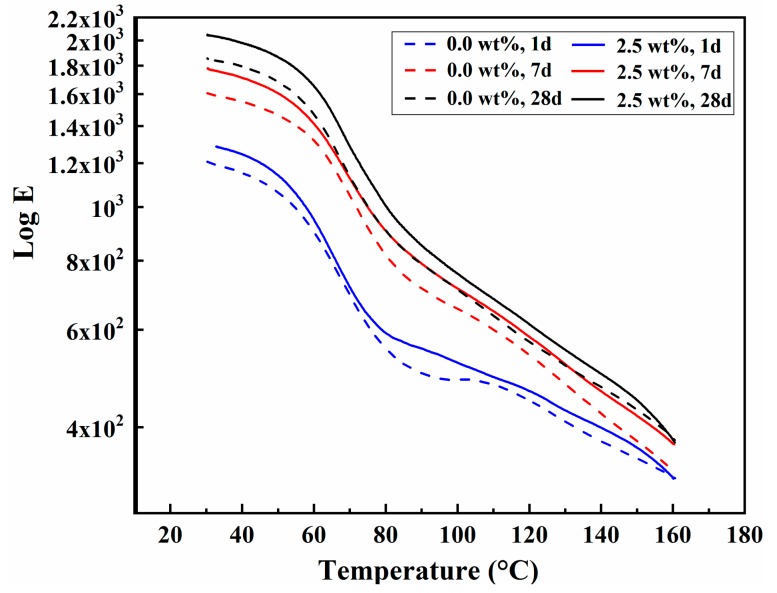
The relationship between storage modulus and temperature of PU, PU/MFA-0.0 wt. %, and PU/MFA-2.5 wt. % specimens at different curing times.

The storage modulus can reflect the hardness of the composites to a certain extent [41]. Figure 9 exhibits the relationship between storage modulus and temperature of PU/MFA-0.0 wt. % and PU/MFA-2.5 wt. % specimens at different curing times. As it can be seen from Figure 9, the storage moduli of PU/MFA-0.0 wt. % and PU/MFA-2.5 wt. % specimens rise steadily as the curing time increases, mainly due to the change in the internal crosslink density of the composites. Besides, the storage modulus value of the PU/MFA-2.5 wt. % specimen is higher than that of PU/MFA-0.0 wt. % at the same curing moment. Abovementioned details further demonstrate that the GPTMS molecule can accelerate the crosslink reaction of the organic PU and MFA, while it promotes the organic reaction sites on the MFA surface and thereby improves the interface properties and crosslink density. Hence, when the material is completely cured inside, the storage modulus value of the PU/MFA-2.5 wt. % specimen rises more notably than that of PU/MFA-0.0 wt. % specimen.

**Figure 10:**
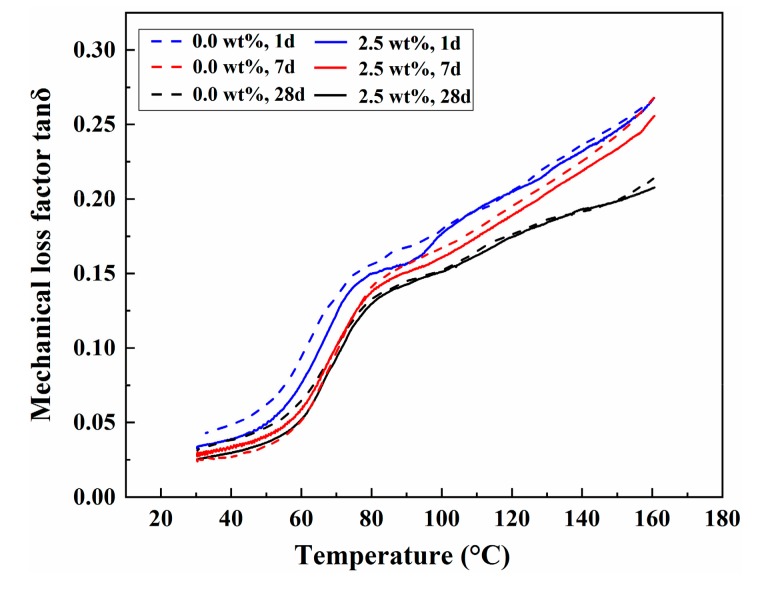
The relationship between loss factors and temperatures of PU, PU/MFA-0.0 wt. %, and PU/MFA-2.5 wt. % specimens at different curing times.

Figure 10 indicates the relationship between the loss factors tan δ and temperatures of PU/MFA-0.0 wt. % and PU/MFA-2.5 wt. % specimens at different curing times. In general, pure PU is a block copolymer consisting of a soft segment and a hard segment. For coal-rock mass reinforcement materials, the glass transition temperature of the hard segment will directly affect the mechanical properties of the PU/MFA composites. It can be seen from Figure 10 that the curve of mechanical loss factor tan δ gradually increases as the temperature *T_g_* raises. This shows that the higher the temperature is, the greater the internal friction of the material gets. The peak value of tan δ corresponds to a temperature between 60 and 90 °C, which can be considered as the glass transition temperature of the hard segment of the material. At this time, the composite materials undergo glass transition, during which the molecular motion energy increases. When the barrier is conquered, the internal motion unit of the molecule is in an activated state and the movement of the segment occurs [42]. Further observation of the curves shows that the glass transition temperatures of the PU/MFA-0.0 wt. % and PU/MFA-2.5 wt. % specimens in the curing process not only become higher but also cover a wider range. This is mainly because as the crosslink density increases, the free volume of the PU/MFA composites decreases and the molecular chain is more constrained. In addition, the average chain length of adjacent crosslink position decreases and further hinders the movement of molecular segment. As a result, the glass transition temperature rises and covers a wider area. Moreover, the glass transition range of PU/MFA-2.5 wt. % specimen is wider than that of PU/MFA-0.0 wt. % at the same curing time, because the modified MFA limits the movement of PU molecular chain and further raises and widens the glass transition temperature. It can be implied that while improving the hardness of the composites, GPTMS has a more positive effect on the improvement of crosslink density.

## 4. Conclusions

In this study, a PU/MFA reinforcement material was prepared by modifying the MFA by 2.5 wt. % GPTMS and compositing it with PU. After the modification, its mechanical properties have been greatly improved. To be specific, its compressive strength, fracture toughness, maximum flexural strength and bond strength increase by 21.6%, 10.1%, 8.8%, and 19.3%, respectively. The results of SEM and DMA further verify that GPTMS promotes the change of MFA surface from hydrophilicity to lipophilicity, eliminates the interfacial difference between organic and inorganic phases, improves the crosslink density and avoids stress concentration. As a result, GPTMS successfully improves the mechanical properties of PU/MFA composites and also positively influences the hardness of the composites. The results of microscopic mechanism analysis show that an end of the GPTMS molecule was cross-linked with MFA surface by hydrolysis, dehydration, and condensation, while the epoxy functional group at the other end was cross-linked with organic matrix through ring-opening reaction. Therefore, the coupling agent GPTMS further broadens the application of PU/MFA composite materials in coal mines. Even in harsh coal mine conditions, it can be effectively applied to the reinforcement of coal-rock mass.

## Figures and Tables

**Table 1:** Components of polyurethane/mesoscopic fly ash (PU/MFA) composites.

Composite	Ingredient	Amount (g)	Manufacturer	Function	Properties
Component A	The polyether polyol (GR4110B)	90 ± 0.1	Shanghai Gaoqiao Petrochemica Co., Ltd.	Reactants	Molecular weight (g/mol): 1500
					Viscosity (mPa.s): 6000–9000
					Hydroxyl value (mg KOH/g): 450
					Moisture (%): ≤0.2
	The polyether polyol (TMN450)	10 ± 0.1	Jiangsu Haian Petrochemical Plant	Reactants	Molecular weight (g/mol): 1300
					Viscosity (mPa·s): 483
					Hydroxyl value (mg KOH/g): 460
					Moisture (%): ≤0.1
	Stannous octanate	0.3 ± 0.02	Shandong Baidu Chemical Co., Ltd.	Catalyzer	Molecular weight (g/mol): 327.56
					Proportion (20 °C): 1.27~1.31
	MFA	140.2 ± 0.1	Huaneng Jinling Power Plant	Aggregate	Moisture (%): ≤0.2
					Particle size (nm): ≤10
					Chloride ion (%): 0.01
	GPTMS	0.7~4.9	Nanjing Nengde Chemical Industry Co., Ltd.	Silane coupling agent	Moisture (%): ≤0.5
	Tris (2-chloro-1-methylethyl) phosphate (TCPP)	1 ± 0.02	Terry New Materials Co., Ltd.	Flame retardant	
Component B	Polymethylene polyphenylene isocyanate (PAPI)	100 ± 0.1	Polyurethane Co., Ltd.	Reactants	Molecular weight (g/mol): 1600
					NCO content (%): 30
					Viscosity (mPa·s): 150–250
					Density (g/mol): 1.25
					Acidity (%): ≤0.05
